# Strength Enhancement of Saline Sludge Through Steel Slag Carbonation Stabilization

**DOI:** 10.3390/ma19194177

**Published:** 2026-09-30

**Authors:** Chunyang Yu, Hongliang Li, Wuping Ran, Zhaofeng Wu, Weizhi Chen

**Affiliations:** 1 School of Traffic and Transportation Engineering, Xinjiang University, Urumqi 830017, China; 2 Xinjiang Key Laboratory of Green Construction and Smart Traffic Control of Transportation Infrastructure, Urumqi 830017, China; 3 Xinjiang Traffic Inspection and Certification Co., Ltd., Urumqi 830009, China; 4 School of Materials Science and Engineering, Xinjiang University, Urumqi 830017, China

**Keywords:** saline sludge, steel slag, carbonation stabilization, strength, composition analysis

## Abstract

The carbonation stabilization method combines the advantages of high efficiency, low carbon emissions, and low alkalinity, making it a green technology with broad application potential for soft soil stabilization. However, in the practice of carbonation stabilization of saline sludge, the impact of soluble salts on the carbonation stabilization system remains poorly understood. This study investigated the mechanical properties and microstructural mineral evolution of sulfate ion (SO_4_^2−^) and chloride ion (Cl^−^) contaminated saline sludge after steel slag carbonation stabilization. The results indicate that the accumulation of Cl^−^ can lead to a significant deterioration in mechanical properties. We hypothesize that this strength reduction is not driven by the formation of chloride-containing expansive Friedel’s salt; instead, it may arise from the hygroscopic nature of chloride salts, which reduces matrix permeability, hinders CO_2_ gas diffusion within the matrix and lowers carbonation efficiency. Notably, XRD and TG-DTG analyses identified no sulfate- or chloride-bearing crystalline phases (e.g., ettringite or Friedel’s salt). We attribute this phase absence to carbonation reactions competing for Ca(OH)_2_ in the stabilized soil’s pore solution, thereby suppressing the crystallization of expansive salt compounds. These findings collectively demonstrate minimal chemical reactivity between the steel slag carbonation system and soluble salts, revealing an intrinsic resistance of carbonated steel slag to salt-induced deterioration.

## 1. Introduction

Each year, over 100 million tons of dredged sludge are discharged from China’s maritime ports to maintain navigation depth, which cannot be directly disposed of due to strict environmental regulations. Chemical stabilization technology provides a viable solution that can convert this sludge into geotechnical materials suitable for road construction, earthfilling projects and water resource conservation engineering [1,2,3]. Chemical stabilization techniques are categorized based on distinct stabilization mechanisms into cement stabilization, alkali-activated stabilization, and carbonation stabilization, among others [4,5,6].

Recent advancements in the domain of soil stabilization have seen a growing interest in the carbonation stabilization method, attributed to its significant merits in both carbon footprint reduction and stabilization efficacy [7,8,9]. However, a major obstacle to the broader application of this method, especially in stabilizing dredged sludge with high moisture content, arises from the current carbonation stabilization techniques. These techniques, reliant on carbonation stabilization agents, are not only costly but also impose stringent soil moisture content requirements. A promising alternative emerges in the form of steel slag—a byproduct of steel manufacturing—boasting a chemical makeup of nearly 40% CaO and 10% MgO, marking it as a potential substitute for carbonation stabilization agents [10]. Capitalizing on the inherent attributes of steel slag, Yu et al. [11] pioneered the steel slag foam drying-carbonation (SSFD-C) method. This innovation employed steel slag pretreatment to extend the applicability of carbonation stabilization technology to the realm of recycling and stabilizing dredged sludge with high moisture content. A comprehensive series of investigations was carried out to explore this novel approach further [12,13,14].

In the SSFD-C method, steel slag serves dual roles: a desiccant during the drying phase, and a stabilization agent during the carbonation stabilization phase for marine dredged sludge. This distinct functionality stems from steel slag’s low hydration activity and high carbonation activity, attributed to its primary mineral, C_2_S [15,16]. Initially, steel slag powder is uniformly mixed with the sludge, enhancing drying efficiency by lowering the slurry’s initial moisture content, facilitating cation exchange, and amplifying the stabilized foam quantity in the slurry—resulting in a nearly 60% reduction in drying time [11]. Post drying, the resultant steel slag-enriched dried soil exhibits reduced volume, mass, and environmental impact, simplifying its transfer, transport, and subsequent resource utilization. When this activated dried soil is rehydrated, compacted, and carbonation-stabilized, a significant strength increase, by 5–8 folds, is observed [11]. The SSFD-C method unveils a novel, low-carbon, and economical pathway for the mitigation, neutralization, and resourceful utilization of dredged sludge.

However, numerous studies addressing marine dredged sludge stabilization have highlighted the significant influence of soluble salts present in the sludge on its stabilization efficacy [17,18]. Particularly, marine dredged sludge often encompasses a high concentration of soluble salts, categorized into SO_4_^2−^, CO_3_^2−^, and Cl^−^ salts, with Cl^−^ salts predominantly found in coastal dredged sludge [19]. The pronounced hygroscopic nature of Cl^−^ salts tends to induce soil body softening, thereby diminishing its bearing capacity [20]. Despite the notable challenges posed by soluble salts, existing literature on cement stabilization aimed at improving the engineering performance of salt-rich soils remains scarce, with reported findings exhibiting inconsistency. Zhang et al. [21] discovered that elevated NaCl salt concentrations deteriorated the strength and stiffness of cement-stabilized soils, linking the detrimental impact on mechanical properties directly to the cement quantity. Similarly, Xing et al. [20] observed that a higher salt content negatively affected cement stabilization efficiency, deducing that Cl^−^ salts exerted a more adverse effect on the strength of cement-stabilized soils compared to SO_4_^2−^ salts. Conversely, Zhou et al. [22] reported enhanced early strength in specimens prepared using seawater (primarily Cl^−^ salts) compared to those utilizing deionized water in a study on incinerated sludge ash and lime-stabilized dredged silt. They concluded that Cl^−^ salts contributed to the stabilization, forming Friedel’s salts as revealed through X-ray diffractometer (XRD), thermogravimetric and derivative thermogravimetric analysis (TG-DTG), and Fourier transform infrared spectroscopy (FTIR) microcosmic composition analysis.

Despite the promising applicability of the carbonation stabilization method for marine dredged sludge stabilization, the impact of soluble salt type and concentration within the sludge on stabilization performance remains unclear. Notably, the mechanisms underpinning the enhancement of soil mechanical properties diverge between steel slag carbonation and traditional cement stabilization. The former strengthens soil structure by filling soil pores and inducing soil compaction through calcium carbonate crystal growth, as depicted in Equations (1)–(4) [23]. Conversely, cement stabilization enhances soil via a formed skeleton from the cementitious product C-S-H generated during cement hydration, illustrated in Equations (5)–(9) [24].

(1) 3CaO·SiO_2_ + 3CO_2_ + nH_2_O→SiO_2_·nH_2_O + 3CaCO_3_

(2) 2CaO·SiO_2_ + 2CO_2_ + nH_2_O→SiO_2_·nH_2_O + 2CaCO_3_

(3) CH + CO_2_→CaCO_3_ + H_2_O

(4) Cx-S-Hy + xCO_2_→SiO_2_·yH_2_O + xCaCO_3_

(5) 2(3CaO·SiO_2_) + 6H_2_O→3CaO·2SiO_2_·3H_2_O + 3Ca(OH)_2_

(6) 2(2CaO·SiO_2_) + 4H_2_O→3CaO·2SiO_2_·3H_2_O + Ca(OH)_2_

(7) 3CaO·Al_2_O_3_ + 6H_2_O→3CaO·Al_2_O_3_·6H_2_O

(8) 4CaO·Al_2_O_3_·Fe_2_O_3_ + 2Ca(OH)_2_ + 10H_2_O→3CaO·Al_2_O_3_·6H_2_O + 3CaO·Fe_2_O_3_·6H_2_O

(9) 3CaSO_4_ + 3CaO·Al_2_O_3_ + 32H_2_O→3CaO·Al_2_O_3_·3CaSO_4_·32H_2_O

Hence, understanding the detrimental mechanisms of strength development in carbonation-stabilized soils by soluble salts can enrich the theoretical foundation in salt-rich soil stabilization. Additionally, analyzing the degradation of mechanical properties in SSFD-C stabilized soils by soluble salts is crucial for enhancing stabilization performance prediction accuracy within the SSFD-C method, especially when treating dredged silt of varying salt types and concentrations. Uniquely, the SSFD-C method entails a drying phase for the steel slag in the slurry before carbonation stabilization, emphasizing interactions between soluble salts and steel slag. The effects and underlying mechanisms of these interactions on strength improvement in SSFD-C stabilized soils warrant further investigation.

This study aims to explore the trajectory and mechanisms underpinning the impact of SO_4_^2−^ and Cl^−^ salts on the strength augmentation of SSFD-C stabilized soils. Assessments were conducted on the unconfined compressive strength (UCS) and strength ratio of carbonation (*R*_CS_) of SSFD-C stabilized soils, with varying concentrations of SO_4_^2−^ and Cl^−^ accumulation, across different carbonation durations. Employing XRD and TG-DTG analysis, the micro-mineral composition and microstructural evolution of steel slag carbonation-stabilized soils were explored under varying soluble salt types and concentrations. These analyses elucidated the pathway and mechanism through which soluble salts undermine the carbonation strength of SSFD-C stabilized soils.

## 2. Materials and Methods

### 2.1. Materials

To attain a more precise analysis of the micro-mineral composition, an artificial soil approach was utilized, comprising 90% quartz powder and 10% kaolinite [25,26]. The quartz powder was sourced from Jiangsu Xinyi City Bright Quartz Sand Factory (Xinyi, China), while the kaolinite was procured from Hunan Guzhang County Shanlin Shiyu Mineral Products Co. (Guzhang, China). Steel slag, with a specific surface area of 117.3 m^2^/kg, was acquired from the basic oxygen furnace (BOF) steel slag of Hebei Jianye Steel Factory (Shijiazhuang, China).

**Figure 1:**
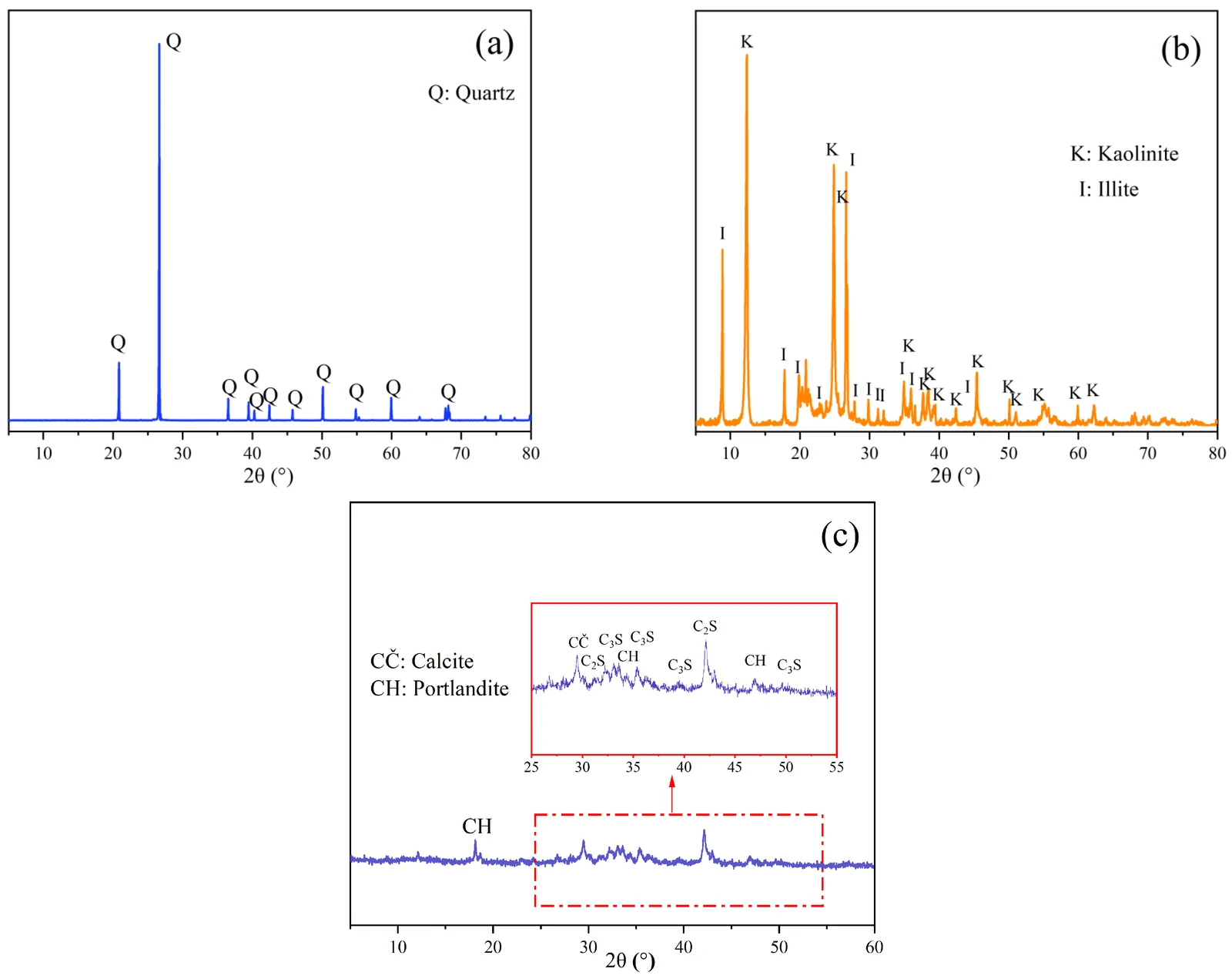
XRD patterns of (**a**) quartz, (**b**) kaolinite, and (**c**) steel slag [13].

The soluble salts employed were chemically pure reagents Na_2_SO_4_ and NaCl, appearing as white crystal particles. The chemical and mineral compositions of the artificial soil raw material and steel slag were examined using XRD and X-ray fluorescence spectroscopy (XRF), with the results shown in Table 1 and Figure 1, respectively. The steel slag adopted in this study is rapidly cooled BOF slag, which contains a substantial amorphous/glassy fraction, a typical characteristic of fast-quenched BOF slag. It should be noted that conventional XRD can only detect crystalline phases such as calcite and portlandite. Fe- and Mn-bearing components may exist in amorphous or poorly crystalline forms and thus cannot be distinguished by XRD. The amorphous fraction was not quantified in the present work, and quantitative characterization of amorphous content will be considered in future research.

### 2.2. Mix Proportions and Sample Preparation

The SSFD-C dried soil (DS) with varying concentrations of salt accumulation was prepared by mixing a salt solution with artificial soil to create a salt slurry, which was then processed through the SSFD-C drying method. As outlined in Table 2, slurries with different salt concentrations were initially prepared. This was achieved by adding a specified volume of salt solution to the artificial soil and thoroughly mixing it until the slurry’s moisture content reached 50%. Here, the salt solution concentration represents the ratio of the weighed salt reagent to the mass of the dried artificial soil. The SSFD-C drying process was detailed in the literature [11], briefly entailing the following steps: a predetermined mass of steel slag was mixed with the salt slurry (with the steel slag admixture amount being the ratio of the mass of dried steel slag to the mass of dried salt-enriched soil). Air bubbles were then introduced for uniform mixing, followed by the placement of the bubble-mixed slurry into convection ovens for drying at a temperature of 40 °C for 48 h. Post drying, the DS was obtained by simple crushing and sieving through a 2 mm mesh.

### 2.3. Test Programs and Methods

Table 2 and Table 3 present the test content and corresponding test programs, respectively. The concentration gradients of NaCl and Na_2_SO_4_ were selected according to the background anion levels in natural marine soft soil reported in [20].

**Figure 2:**
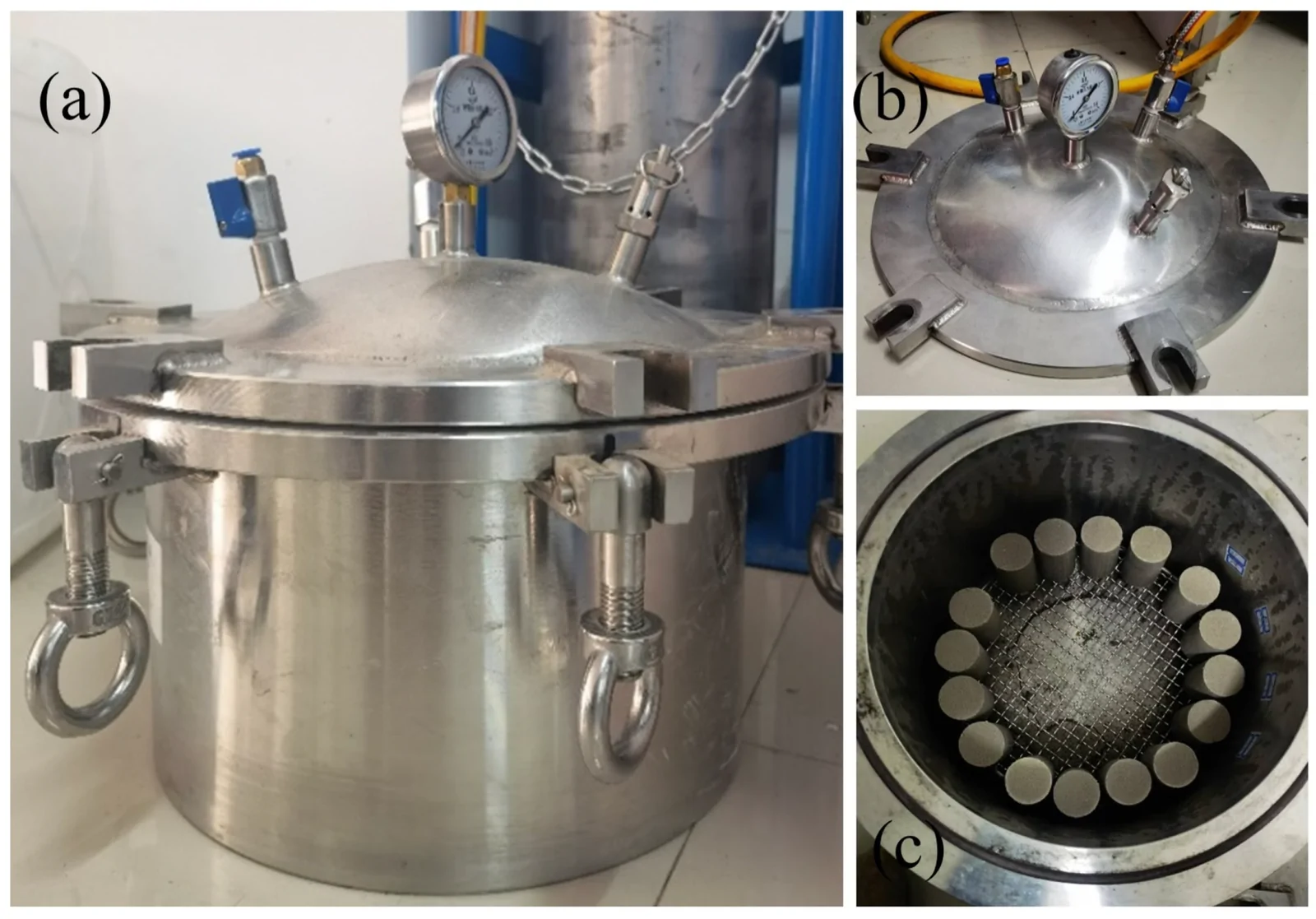
Photos of the carbonation chamber device: (**a**) An overview of the entire carbonation chamber device; (**b**) the cover of the device; and (**c**) the bottom of the device.

Referring to Table 3, deionized water was weighed and mixed with DS. The DS specimens were prepared by static compaction in a cast iron mold to form soil columns with a diameter of 50 mm and height of 100 mm under a forming load of 2 kN. The SSFD-C stabilized soil, denoted as DS-C, was obtained by placing the DS specimen into the carbonation reactor (shown in Figure 2) for carbonation. Carbonation was performed using 99.9% pure CO_2_ at a pressure of 0.2 MPa. All specimens were carbonated within 2 h after demolding. UCS tests were performed within 24 h after carbonation was finished. Additionally, the MS group, a direct mixture of dried salt, artificial soil and steel slag, was prepared to assess how the drying process enhances the interaction between salt and steel slag in the SSFD-C method. MS specimens were prepared using the same compaction method.

The evaluation of UCS was conducted in alignment with the specifications laid out in Standard ASTM D4219-08, employing a strain rate of 0.5 mm/min. An average value along with the standard deviation was calculated from the UCS of three concurrent specimens within each set. Following the completion of UCS assessments, segments from the central portion of the fragmented specimens were selected, subjected to a vacuum drying process at 60 °C for a duration of 24 h, subsequently ground down and sieved through an 80 μm mesh to prepare samples for pH assessments and additional mineral composition analyses.

To quantify the enhancement in bearing capacity attributable to carbonation stabilization across varying soil types, a metric termed *R*_CS_ was introduced. *R*_CS_ is derived from the ratio of the UCS post-carbonation treatment to the pre-carbonation strength. The formula employed for this calculation is expressed as follows:

(10) $$R_{CS}=\frac{{UCS}_{DS-C}}{{UCS}_{DS}}$$

where UCS_DS-C_ symbolizes the unconfined compressive strength of a DS-C specimen, representing the post-carbonation stabilization strength; while UCS_DS_ denotes the unconfined compressive strength of a DS specimen, signifying the pre-carbonation stabilization strength.

pH assessments were carried out utilizing a PHS-3E pH meter (Shanghai Precision Scientific Instrument Co., Ltd., Shanghai, China). A mixture comprising 2 g of powder and 20 mL of deionized water was shaken in a 25 mL test tube for a span of 5 min, with pH evaluations completed within the subsequent hour. The compositional examination of five distinct types of DS, DS-C, and MS was performed using a Bruker D8 ADVANCE X-ray diffractometer (Karlsruhe, Germany), employing CuKα_12_ radiation (K = 0.154 nm). The operational settings for the X-ray source were fixed at 40 kV voltage and 40 mA current. The morphology of carbonation products in the two types of DS-C were observed via SEM (TESCAN MIRA, Brno, Czech Republic) at an accelerating voltage of 3 kV.

## 3. Results

### 3.1. Optimal Moisture Content for Carbonation Stabilization (C-OMC)

As explained aforementioned, C-OMC represents the ideal moisture content at which carbonation stabilization, facilitated by steel slag, achieves maximal effectiveness. The efficacy of carbonation stabilization is closely tied to the soil’s moisture content due to the process’s reliance on gaseous interactions. When soil moisture content is low, the dissolution of CO_2_ and the interaction with Ca^2+^ ions from steel slag are inhibited. On the other hand, an overly high moisture content suppresses CO_2_ gas diffusion through the soil and leads to an increase in void spaces within the stabilized soil, consequently reducing its strength [14].

**Figure 3:**
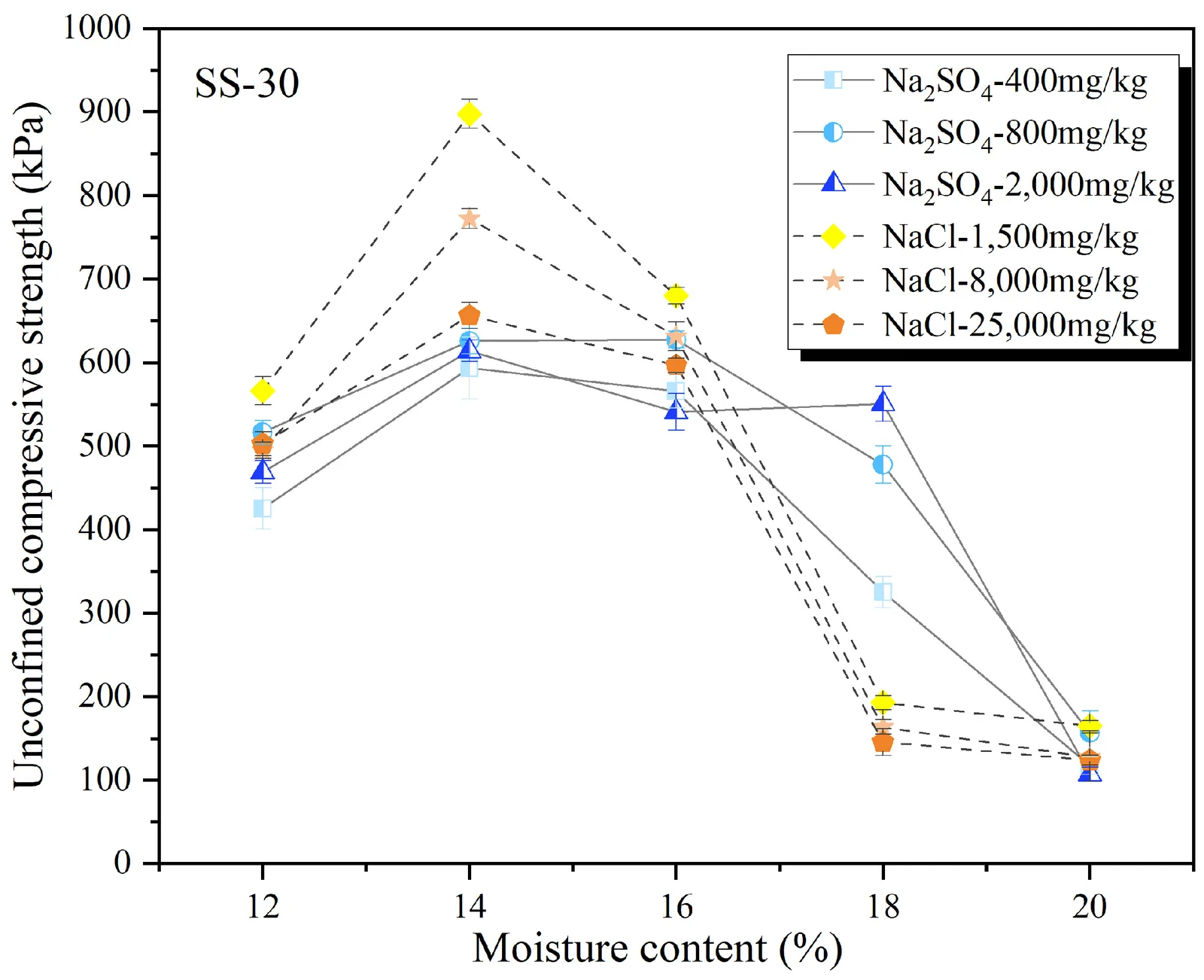
Variation in the UCS of DS specimens with different moisture contents: impact of SO_4_^2−^ and Cl^−^ accumulation concentrations.

Figure 3 presents the UCS results of DS-C specimens obtained through the C-OMC testing, shedding light on the effects of salt type and accumulation concentration. The findings reveal a trend where the UCS of DS-C specimens, with varying concentrations of SO_4_^2−^ and Cl^−^, initially increases before declining with escalating moisture content, reaching a peak carbonated strength at 14% moisture content.

Moreover, a broader range of effective carbonated moisture content between 12% and 18% was observed for different concentrations of SO_4_^2−^ accumulation, while a narrower effective moisture content range between 12% and 16% was noted for varying concentrations of Cl^−^ accumulation. This illustrates that Cl^−^ accumulation heightens the water sensitivity of DS specimens concerning carbonation stabilization. A notable decrease of about 75% in the UCS of DS-C specimens was recorded when the moisture content for carbonation stabilization was raised from 16% to 18%, essentially indicating a failure in carbonation stabilization. This scenario was associated with the softening of Cl^−^ accumulated DS specimens when molded at 18% moisture content for carbonation stabilization. Previous studies have reported that Cl^−^-rich salt soils exhibit a lower plastic limit moisture content compared to non-salt-rich soils, implying that Cl^−^-rich salt soils can transition to a liquid state at relatively lower moisture contents [17].

### 3.2. Carbonation-Induced Strength Enhancement

**Figure 4:**
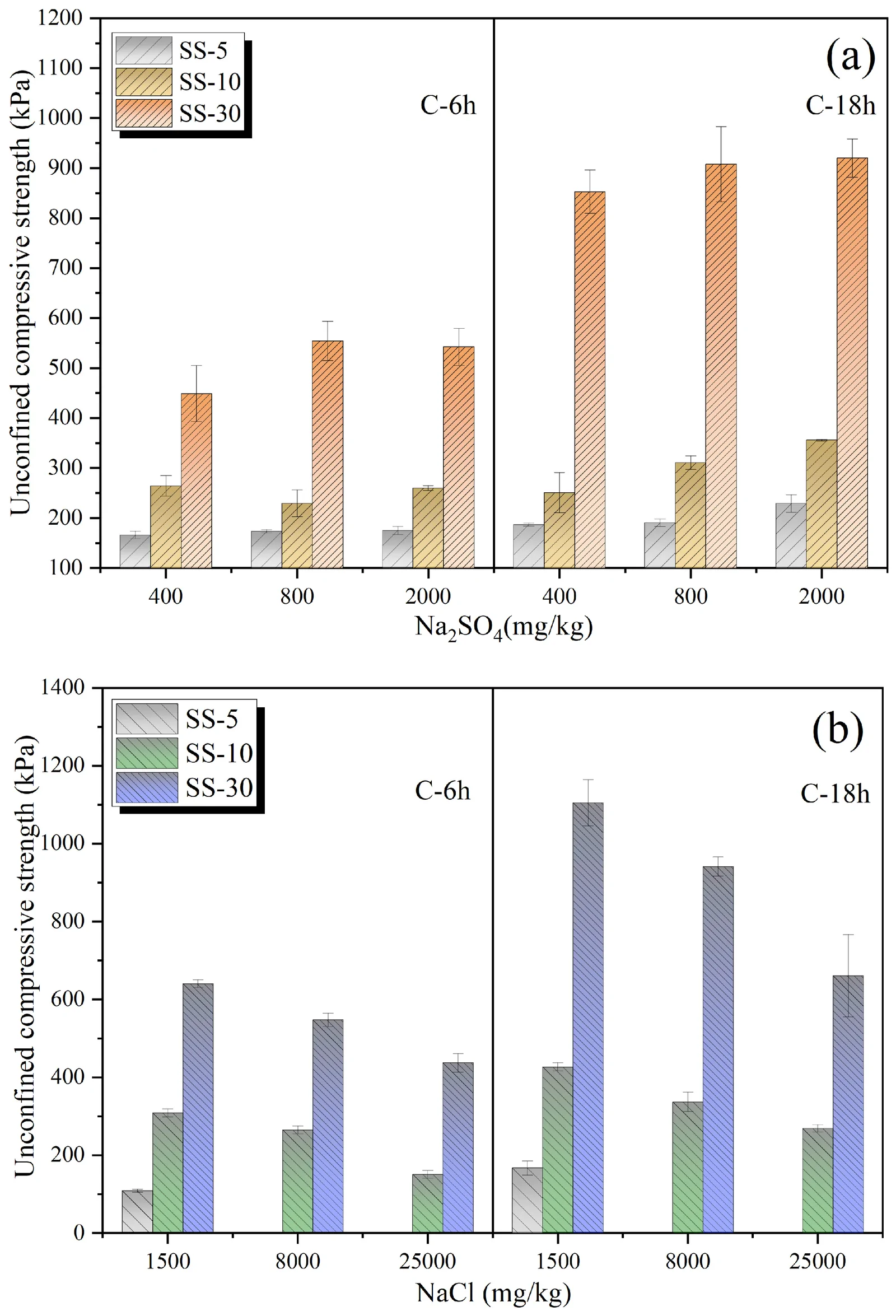
UCS of DS-C specimens (C-6 h and C-18 h) influenced by different salt types: (**a**) Na_2_SO_4_ and (**b**) NaCl.

Figure 4 illustrates the UCS of DS-C specimens influenced by the type and accumulation concentration of salts. For DS specimens with SO_4_^2−^ accumulation, increasing steel slag dosage raised the strength of DS-C specimens after 6 h of carbonation. Within the tested range, variations in the accumulated SO_4_^2−^ concentration did not produce an appreciable change in specimen strength. Extending the carbonation stabilization duration to 18 h continued to show strength enhancement with an increased percentage of steel slag, although there was a slight upward trend in strength as the SO_4_^2−^ accumulation concentration increased.

Conversely, for DS specimens with Cl^−^ accumulation, the strength of DS-C specimens rose with the escalation in steel slag percentage during a carbonation stabilization period of 6 h. However, alterations in Cl^−^ accumulation concentration markedly affected the strength of DS-C specimens, manifesting as a notable decline in strength with increased Cl^−^ accumulation concentration. Upon extending the carbonation stabilization duration to 18 h, although the strength of DS-C specimens in this group surpassed that of specimens carbonated for 6 h, a pronounced decline in strength with escalating Cl^−^ accumulation concentration remained evident. Within the respective concentration ranges tested, Cl^−^ concentration increase leads to progressive strength loss in DS-C specimens, while the strength variation induced by SO_4_^2−^ is limited within its test range.

Specifically, observations during testing revealed that at a lower steel slag content (5 wt%), the DS specimens exhibited carbonation strength only with a lower Cl^−^ content (1500 mg/kg), irrespective of whether the carbonation and stabilization time was 6 h or 18 h. However, a higher cumulative concentration of Cl^−^ led to the emergence of transverse cracks in the DS specimens post-carbonation, resulting in stabilization failure. This occurrence of transverse cracks can be attributed to two primary factors: firstly, the solid–liquid conversion process involving soluble salts was found to either bind some water molecules forming solid crystals or release these molecules into the soil. Cl^−^ exhibited a higher propensity to bind free water compared to SO_4_^2−^, acting as water of crystallization, and subsequently releasing these water molecules upon rehydration, thereby increasing free water content. Secondly, the intensive maintenance of carbonation stabilization within the carbonation kettle was identified as a contributing factor. The carbonation reaction, being exothermic, released a substantial amount of heat instantaneously, which intensified the exchange of water vapor within the matrix of SS-5 specimens. The high-pressure migration of water vapor spurred the development of cracks.

In the study by Xing et al. [20] examining cement utilization for stabilizing salt-rich soil, a relationship was identified between the strength of stabilized soil and the total quantity of hydration products C-S-H and C-A-H. It was deduced that escalating the cumulative concentration of Cl^−^ salts contributed to the weakening of stabilized soil strength by affecting the cement’s hydration process, thereby reducing the total quantity of hydration products (C-S-H and C-A-H). Thus, this paper hypothesizes that increasing the carbonation products led to an overall strength increase in the stabilized salt DS specimens when the carbonation duration was extended from 6 to 18 h for the Cl^−^ accumulated DS-C specimens. Irrespective of the carbonation duration, the strength reduction in salt DS specimens with escalating Cl^−^ accumulation concentration is attributed to the strong hygroscopicity of Cl^−^ salts. This results in the relative carbonation moisture content surpassing the C-OMC upon rehydration of DS, leading to a reduction in carbonation efficiency, a reduction in the total amount of carbonation products, and consequently, a strength decrease. This hypothesis will be further explored in the micro-composition analysis that follows.

In addition to the DS-C’s unconfined compressive strength, the DS unconfined compressive strength pre-carbonation should be considered to evaluate the contribution of carbonation stabilization to the strength enhancement of different soil compositions [11]. Table 4 displays the unconfined compressive strength of DS specimens, influenced by the type and accumulation concentration of salts. As observed in Table 4, before carbonation, the type and accumulation concentration of salts exerted some influence on the unconfined compressive strength of DS specimens.

For SO_4_^2−^-accumulated DS specimens, the strength increased with increasing steel slag content, which is attributed to the skeleton effect provided by the coarser steel slag particles, whereas the SO_4_^2−^ accumulation concentration had little effect. For the DS specimens with Cl^−^ salt accumulation, their strength also rose with the increment of steel slag content in DS, but the change in Cl^−^ salt accumulation concentration appeared to significantly affect their strength, which was reflected in the following: with the escalation of Cl^−^ salt accumulation concentration, DS specimens’ strength decreased dramatically. This is related to the hygroscopicity of chloride salt; with the rise in cumulative concentration of chloride salt, after adding water again to the molding, the crystalline Cl^−^ dissolves and releases a large amount of free water, leading to the softening of the specimen and the strength degradation.

**Figure 5:**
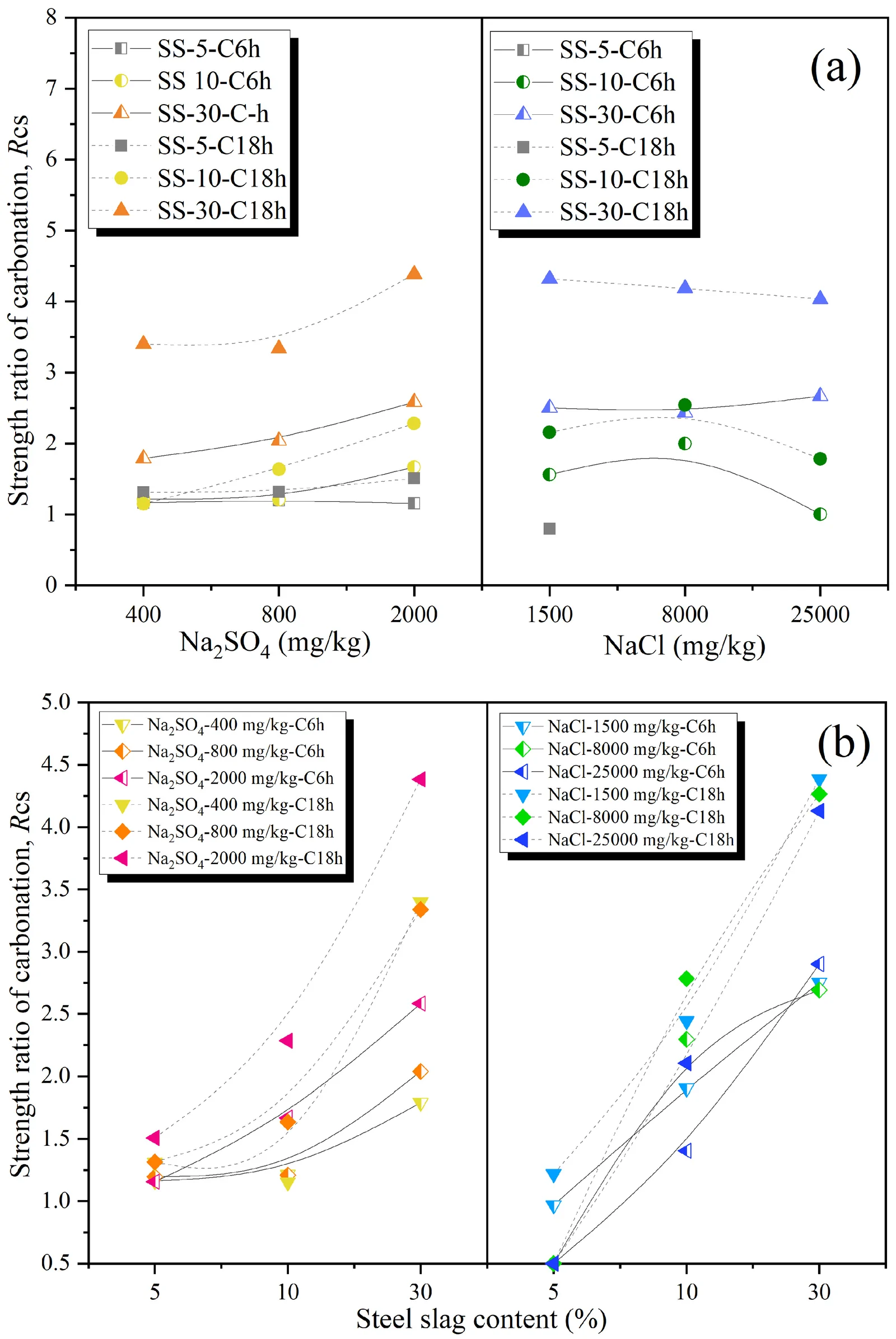
*R*_CS_ of DS-C specimens influenced by different salt types: (**a**) Na_2_SO_4_ and NaCl and by (**b**) steel slag content.

The *R*_CS_ values for DS-C specimens, reflecting the influence of salt type and accumulation concentration, are depicted in Figure 5, having accounted for the unconfined compressive strength of DS before carbonation. For DS specimens with SO_4_^2−^ accumulation, a minor increase in *R*_CS_ is noted as the SO_4_^2−^ accumulation concentration rises, whereas for those with Cl^−^ accumulation, a slight decline in *R*_CS_ is observed with an increasing Cl^−^ accumulation concentration. The trend in *R*_CS_ for DS-C specimens, under varying salt types and accumulation concentrations, aligns with the findings regarding unconfined compressive strength illustrated in Figure 4.

Moreover, the trends in *R*_CS_ for DS-C specimens, with increasing concentrations of SO_4_^2−^ or Cl^−^ accumulation, do not exhibit a significant correlation with the quantity of steel slag admixture and the duration of carbonation maintenance. Further, in DS specimens with SO_4_^2−^ accumulation, when the steel slag content in DS is minimal (5%), the alteration in SO_4_^2−^ concentration does not significantly impact the *R*_CS_ of DS-C specimens. However, when the steel slag content in DS is substantial (30%), the effect of changing SO_4_^2−^ concentration on the *R*_CS_ of DS-C specimens amplifies.

On the other hand, for DS specimens with Cl^−^ accumulation, the impact of changing Cl^−^ concentration on DS-C specimen *R*_CS_ is conspicuous when the DS contains a minimal amount of steel slag (5%), whereas the impact of changing Cl^−^ concentration on DS-C specimen *R*_CS_ is subtle when the DS contains a substantial amount of steel slag (30%). This indicates that, for DS specimens with Cl^−^ accumulation, the strength of DS-C specimens with low steel slag dosing is susceptible to the concentration of Cl^−^ salts, while high steel slag dosing is beneficial in mitigating the adverse effects of Cl^−^ salts on their strength.

### 3.3. Microscopic Mineral Analysis

**Figure 6:**
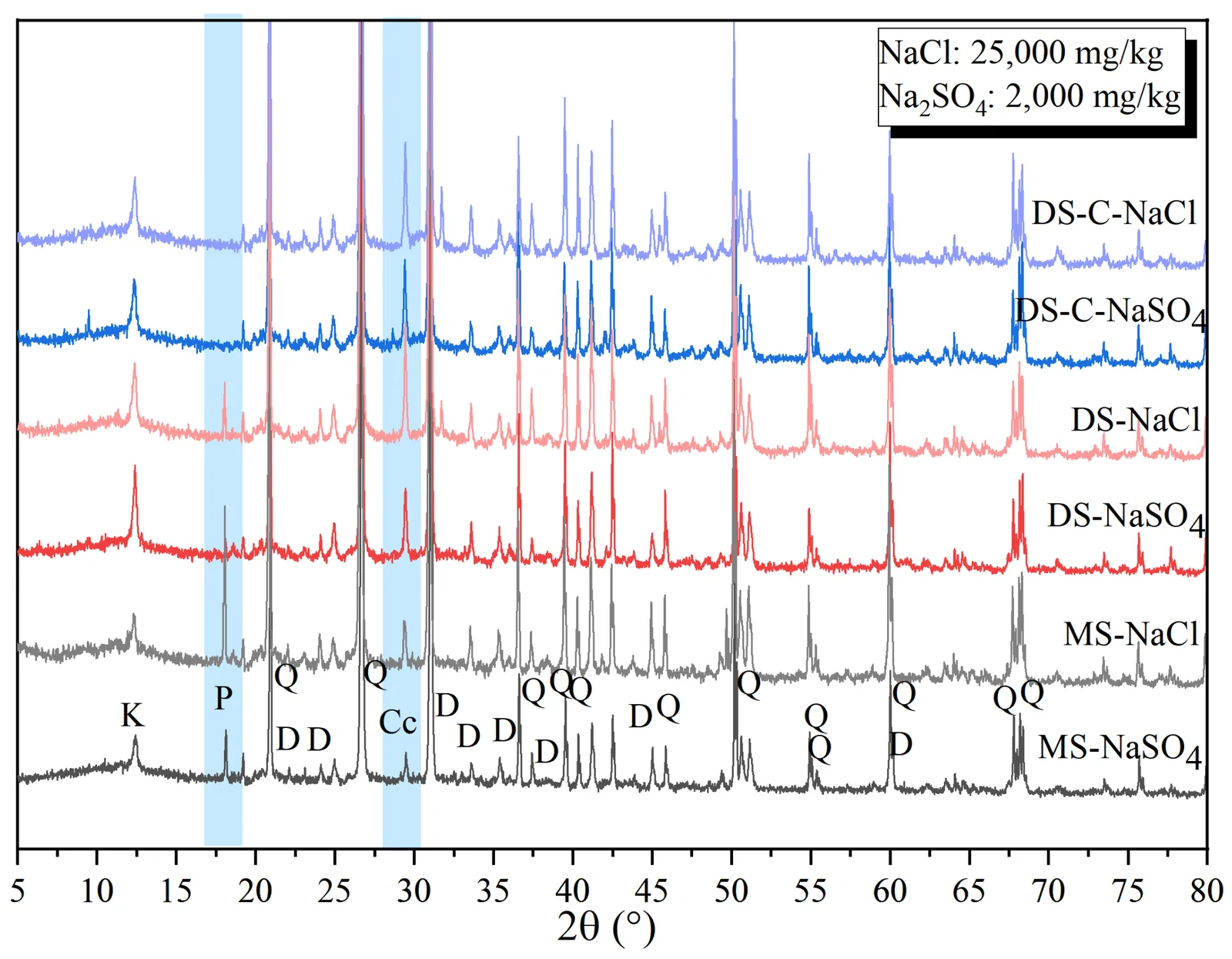
XRD patterns of DS, DS-C, and MS specimens influenced by the salt type and accumulation concentration.

Figure 6 presents the XRD patterns of MS, DS, and DS-C specimens under varying salt types and accumulation concentrations. It was found that the mineralogical composition of MS specimens was relatively unaltered under both SO_4_^2−^ and Cl^−^ accumulations, primarily comprising kaolinite (Kaolinite, K), Portlandite (Ca(OH)_2_, CH), quartz (SiO_2_, Q), calcite (CaCO_3_, Cc), and dolomite (CaMg(CO_3_)_2_, D). When compared to the SO_4_^2−^ accumulated MS specimen, the diffraction peak at CH was more pronounced for the Cl^−^ salt-accumulated MS specimen, potentially due to the accelerated dissolution of steel slag by Cl^−^ salt.

Upon examining the DS specimens, a notable reduction in the diffraction peaks at CH compared to MS specimens was observed for both salt types. Concurrently, an elevated diffraction peak intensity at Cc was noted for both salt-accumulated DS specimens relative to MS specimens, suggesting that aerial carbonation of CH transpired during the drying process. Moreover, in DS-C specimens, the diffraction peak at CH was absent for both salt-accumulated DS-C specimens compared to DS specimens, indicative of CH’s conversion to Cc due to carbonation [27].

Interestingly, for the MS, DS, and DS-C specimens with Cl^−^ salt accumulation, Friedel’s salt (3CaO∙Al_2_O_3_∙CaCl_2_∙10H_2_O) diffraction peaks were undetected at 2θ of 11.5º versus 55.1º. Numerous studies on cement stabilization have demonstrated that Friedel’s salt significantly influences the strength of salt-rich soils. The formation of Friedel’s salt is depicted in Equation (11):

(11) 3CaO·Al_2_O_3_ + Ca^2+^ + 2Cl^−^ + 10H_2_O→3CaO·Al_2_O_3_·CaCl_2_·10H_2_O

It is postulated that CH underwent carbonation, competing for the reaction and thereby thwarting the synthesis of Friedel’s salt. This assumption will be further explored in the subsequent TG-DTG analysis.

**Figure 7:**
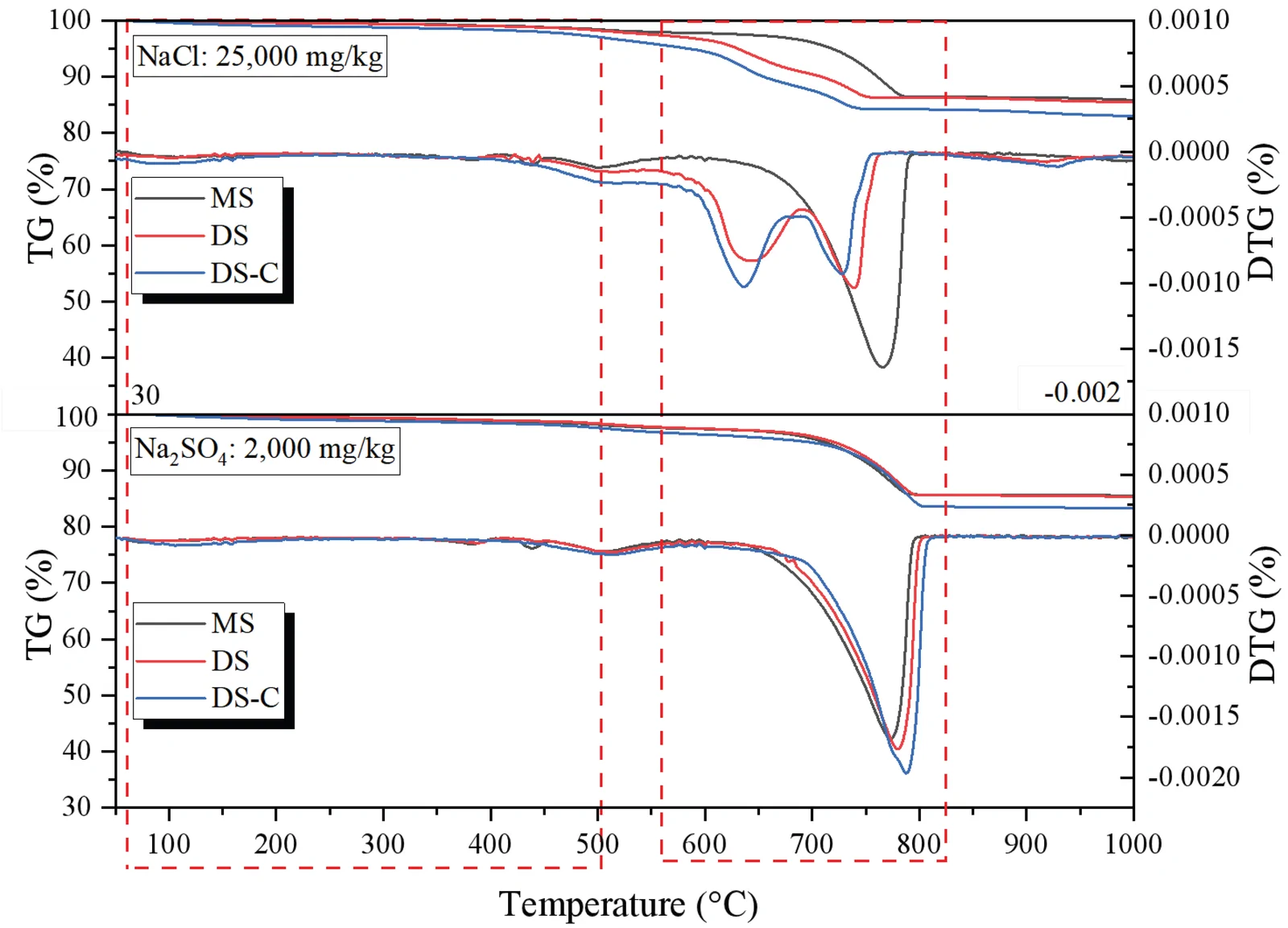

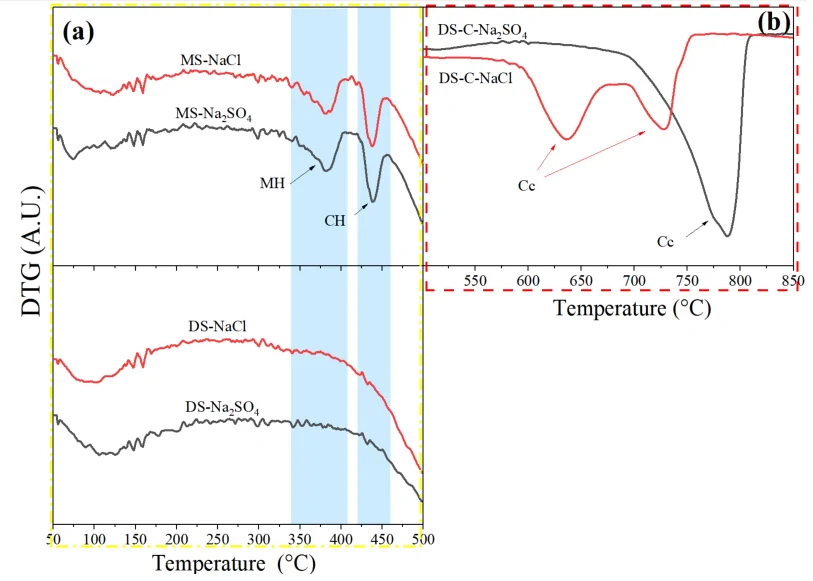
TG-DTG analysis of DS, DS-C, and MS specimens influenced by the salt type and accumulation concentration. (**a**) Local DTG curve in the temperature range of 50–500 °C; (**b**) Local DTG curve in the temperature range of 500–859 °C.

Figure 7 illustrates the TG-DTG analysis of MS, DS, and DS-C specimens subjected to different salt species and accumulation concentrations. Figure 7a displays a localized DTG plot within the temperature range of 50–500 °C. For both MS specimens with salt accumulation, weight loss peaks emerged within the temperature ranges of 350–400 °C and 425–450 °C. It is hypothesized that the weight loss observed between 350–400 °C as well as 425–450 °C may be attributed to the decomposition of Mg(OH)_2_ (MH) and CH [11]. Furthermore, the literature indicates that the thermal decomposition temperature range for Friedel’s salt is between 230–410 °C [22], partially coinciding with the decomposition temperature for MH. Nevertheless, a similar weight loss at 350–400 °C and 425–450 °C was noted for MS specimens under both salt influences; thereby, it can be inferred that the weight loss at 350–400 °C is not due to Friedel’s salt decomposition, supporting the XRD findings presented in Figure 6, where none of the chloride-salt-accumulated MS, DS, and DS-C specimens exhibited Friedel’s salts. Additionally, the vanishing of CH and MH in DS specimens after the SSFD-C drying process might be associated with the enhanced solubility of these salts, rendering them more prone to carbonation and atmospheric consumption during the drying phase.

Figure 7b displays localized DTG plots within the temperature span of 500–850 °C. For DS-C specimens with SO_4_^2−^ accumulation, pronounced weight loss peaks at 700–820 °C were due to calcium carbonate decomposition, while DS-C specimens with Cl^−^ accumulation exhibited double weight loss peaks within 600–675 °C and 680–750 °C temperature ranges. Two reasons are hypothesized for these double weight loss peaks being related to CaCO_3_ decomposition:

(1) Initially, weight loss within the stable range of 600–675 °C attributed to MgCO_3_ decomposition can be rejected. Literature reports that the decomposition temperature of MgCO_3_ generally ranges between 650 and 750 °C [28]. However, if the weight loss within 600–675 °C was due to MgCO_3_ decarbonation, SO_4_^2−^ accumulated DS-C specimens should exhibit weight loss within this range, contrasting with the test results. Hence, the weight loss within 600–675 °C is not due to MgCO_3_ decomposition. The absence of weight loss from MgCO_3_ decomposition in both salt-accumulated DS-C specimens is related to lower MH content in their DS specimens.
(2) Secondly, double weight loss peaks at 600–675 °C and 680–750 °C have been observed in DS specimens with accumulated Cl^−^ salts. It is proposed that Cl^−^ salts significantly enhance CH solubility compared to SO_4_^2−^ salts, and the weight loss at 600–675 °C is presumed to be the outcome of a large amount of poorly crystallized CaCO_3_ generated by air carbonation due to the enhanced CH solubility in the DS specimens, whereas the carbonation stabilization reaction produces better-developed CaCO_3_ crystals that decompose at higher temperatures, thus resulting in the double weight-loss peaks. Additionally, for Cl^−^-accumulated DS-C specimens, both CaCO_3_ weight loss peak temperatures were lower than those of the SO_4_^2−^ accumulated DS-C specimens, and the peak areas of the DTG curves were smaller, indicating that Cl^−^ salts hinder the carbonation reaction more significantly than the SO_4_^2−^ salts, producing fewer carbonation products, and consequently, a lower intensity. This micro-compositional analysis elucidates that the *R*_CS_ values of DS-C specimens exposed to Cl^−^ salts are lower than those of counterparts subjected to SO_4_^2−^ salts.

**Figure 8:**
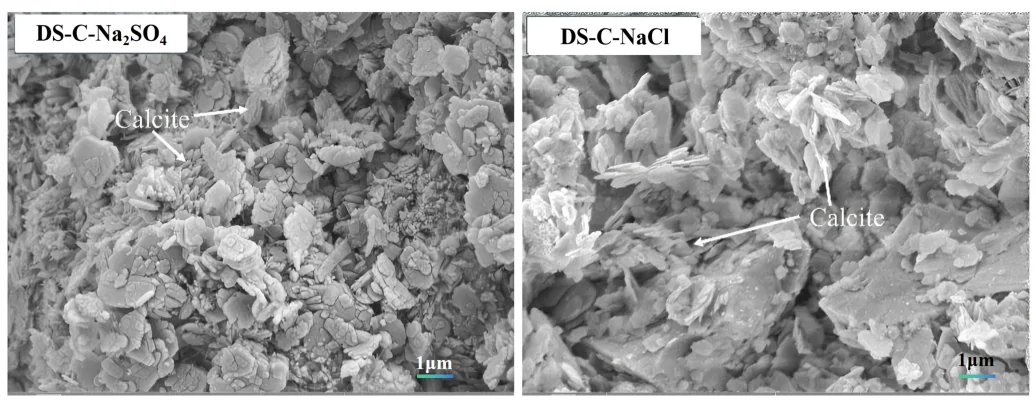
The SEM images of DS-C specimens influenced by the salt type.

Figure 8 presents SEM images of DS-C samples exposed to different salts. The results indicate that under sulfate exposure, the aggregation of calcium carbonate crystals in the DS-C samples was more pronounced, though the crystals were smaller in size. Conversely, under chloride exposure, the aggregation was less intense, and the crystals exhibited a flake-like morphology. This is likely due to two factors: firstly, the chloride salts accelerated the dissolution of Ca^2+^ from the steel slag, allowing the calcium carbonate crystals more space to grow and disperse; secondly, the lower carbonation degree in the DS-C samples hindered crystal development, resulting in thinner, flake-like structures. Notably, no Friedel’s salts were detected in the DS-C samples influenced by chloride salts, consistent with the XRD and TG-DTG results.

## 4. Discussion

This study investigates the effects of soluble salts within a novel carbonation solidification system. A key observation is that, even with prolonged carbonation duration, the strength of DS-C specimens with accumulated Cl^−^ gradually decreases as Cl^−^ concentration rises. This indicates that strength degradation of SSFD-C stabilized soil induced by Cl^−^ accumulation does not originate from expansion associated with Friedel’s salt formation and accumulation.

We hypothesize that the hygroscopic property of chloride salts increases the effective moisture content beyond the C-OMC and thereby reduces CO_2_ diffusivity. Alternative contributing mechanisms, including modified compaction characteristics, ionic-strength effects on clay fabric, changes in pore-solution chemistry, efflorescence, and salt crystallization stresses, cannot be ruled out.

Conventional cement-based solidification faces a well-documented trade-off between chemical salt fixation and long-term salt corrosion resistance. To mitigate excessive expansion, several researchers have proposed improved strategies that forgo chemical salt fixation. Typical approaches include: (1) using cement with a lower C_3_A content, such as special cement resistant to sulfur aluminate erosion [29]; (2) adding nano-SiO_2_ to consume free Ca(OH)_2_ in solidified soil through pozzolanic reaction [30]; (3) employing calcium-free alkali activators for alkali-activated stabilization, such as using NaOH or water glass as alkali activators [31]. However, although special cement and nanomaterials can effectively enhance stabilization performance, their high costs limit large-scale application, and the use of calcium-free alkali activators may pose risks of soil and groundwater pollution.

Friedel’s salt was not detected via XRD and TG-DTG microstructural analyses under the tested conditions of this steel slag carbonation stabilization system. The absence of diagnostic XRD peaks or distinct TG-DTG events does not prove that Friedel’s salt is entirely absent; this phase may exist below the detection limit or with poor crystallinity and thus escape identification. Furthermore, reactive aluminum within this system is limited: the steel slag contains only 3.84% Al_2_O_3_, and the kaolinite-bearing matrix further reduces reactive Al availability, which may limit Friedel’s salt formation and constrain the interpretation of this non-detection result. Accordingly, this observation provides a tentative indication of the long-term salt-resistant and environmentally friendly performance of the steel slag carbonation stabilization system, rather than serving as definitive proof.

It should be emphasized that all statements regarding long-term salt resistance, low carbon emissions, environmental compatibility, minimal chemical reactivity, and broad field suitability are treated as working hypotheses for future investigation, rather than conclusions supported by the present dataset. This study only evaluates short-term UCS performance under accelerated carbonation for 6 h or 18 h. Long-term strength retention, wet–dry cycles, salt cycling, immersion behavior, volumetric swelling, and leaching characteristics were not examined in the current test program. Rigorous assessment of environmental compatibility would require supplementary chemical characterization, elutriate testing, ecotoxicity assays, and statistical evaluation, as recommended in prior literature [32]. Further multi-scale and long-term experiments are therefore required to validate these hypothesized advantages before field application.

For stabilization approaches that abandon chemical salt fixation, the migration pathways of soluble salts within the soil matrix remain unclear, which will be the focus of future research on this carbonation stabilization method.

Additionally, the quartz–kaolinite model soil used in this study cannot fully replicate the particle-size distribution, organic matter content, indigenous carbonates, pore-water chemistry, contaminants and inherent heterogeneity of natural marine mud. Future work may examine the influences of degradable organic matter and microplastics on the performance of the SSFD-C technique [33].

## 5. Conclusions

This study examined how SO_4_^2−^ and Cl^−^ accumulation affects the mechanical properties of carbonation stabilization, contributing a complementary theory to the study of chemically stabilized salt-rich soils. Under salt influence, the variation rule of the ratio of UCS and *R*_CS_ of SSFD-C stabilized soils was investigated. The comparative study with the conventional carbonation method, along with the analysis of the evolution of the mineral composition during the drying and carbonation processes through XRD and TG-DTG, revealed the intrinsic mechanism of the influence of SO_4_^2−^ and Cl^−^ on the stabilization efficiency of SSFD-C. Key findings include:

1. Compared to SO_4_^2−^ accumulation, Cl^−^ accumulation increases the water sensitivity of DS specimens during carbonation stabilization, leading to a roughly 75% decrease in the UCS of DS-C specimens when the moisture content for carbonation stabilization rises from 16% to 18%, rendering carbonation stabilization largely ineffective.
2. For SO_4_^2−^ accumulated DS specimens, *R*_CS_ exhibits a minor increase trend with the escalation of SO_4_^2−^ accumulation concentration; for Cl^−^-accumulated DS specimens, *R*_CS_ shows a minor decline trend with the escalation of Cl^−^ accumulation concentration. Moreover, for Cl^−^-accumulated DS samples, the carbonation strength of DS with low slag content is sensitive to the concentration of Cl^−^ salt, while a high percentage of slag in DS helps mitigate the adverse effect of Cl^−^ salt on its carbonation strength.
3. The deterioration mechanism of the strength of SSFD-C stabilized soils by Cl^−^ salt accumulation is primarily linked to Cl^−^ salts causing the relative moisture content of carbonation stabilization to exceed the C-OMC through the transformation of water of crystallization, resulting in a notable decrease in carbonation efficiency. The amount of salt crystallization water release should be considered in the design of carbonation stabilization moisture content.
4. The implications of these findings extend to the broader understanding of soil stabilization techniques, particularly under the influence of different salt accumulations. This study underscores the importance of factoring in the type and concentration of salts present in the soil when designing carbonation stabilization processes, to ensure optimal mechanical performance and longevity of stabilized soils. Future research may delve deeper into exploring the interactive effects of different salt types and their concentrations, as well as varying carbonation conditions, to further refine and enhance the efficiency and effectiveness of carbonation stabilization methodologies for salt-rich soils.

## Figures and Tables

**Table 1:** Chemical compositions of raw materials (wt%) [9].

Sample	CaO	SiO_2_	Fe_2_O_3_	Al_2_O_3_	MgO	Na_2_O	K_2_O	MnO
Quartz	-	99.98	-	-	-	-	-	-
Kaolinite	0.22	54.82	1.69	37.95	0.21	-	3.01	0.02
Steel slag	39.01	14.46	23.41	3.84	7.28	-	-	7.31

**Table 2:** Proportion of artificial soil.

Quartz Powder (wt%)	Kaolinite (wt%)	Na_2_SO_4_ (mg/kg)	NaCl (mg/kg)
90	10	400	-
		800	-
		2000	-
		-	1500
		-	8000
		-	25,000

Note: The dry artificial soil consists of quartz powder (90 wt%) and kaolinite (10 wt%). The concentration of Na_2_SO_4_ or NaCl is defined as the mass of Na_2_SO_4_ or NaCl reagent divided by the mass of dry artificial soil (mg/kg dry soil).

**Table 3:** Test program.

Study Content	Moisture Content (wt%)	Steel Slag Content (wt%)	Carbonation Time (h)	Test Subject	Test Content
1. Effect of salt types and accumulation concentrations on the moisture content of DS during optimal carbonation cure.	12, 14, 16, 18, 20	30	6	DS-C	UCS
2. Impact of salt types and accumulation concentrations on the strength of DS carbonation stabilization.	Optimal moisture content for carbonation (C-OMC)	5, 10, 30	6, 18	DS, DS-C	UCS
3. Examination of mineral composition subject to variations in salt types and accumulation concentrations.		30	6, 18	MS, DS, DS-C	XRD, TG-DTG

Note: Steel slag content = mass of dry steel slag powder/mass of dry saline soil (wt%); moisture content = mass of water/mass of dry soil (wt%). Note that C-OMC denotes the optimal moisture content for the most efficient carbonation stabilization [11]. At this moisture content, chemical reactions among CO_2_, steel slag and soil proceed optimally, achieving the expected improvement in soil strength and stability.

**Table 4:** The UCS of DS specimens influenced by the salt type and accumulation concentration.

Salt Type	Salt Concentration (mg/kg)	UCS (kPa)		
		SS-5	SS-10	SS-30
Na_2_SO_4_	400	143	218	251
	800	145	190	272
	2000	152	156	210
NaCl	1500	210	198	256
	8000	148	133	225
	25,000	79	151	164

## Data Availability

The original contributions presented in the study are included in the article, further inquiries can be directed to the corresponding author.
